# A physiologically inspired model for global remapping in the hippocampus

**DOI:** 10.1186/1471-2202-12-S1-P194

**Published:** 2011-07-18

**Authors:** Axel Kammerer, Alexander Mathis, Martin Stemmler, Andreas Herz, Christian Leibold

**Affiliations:** 1 Division of Neurobiology, Ludwig-Maximilians-Universität München, 82152 Martinsried, Germany; 2 Bernstein Center for Computational Neuroscience Munich, University, 82152 Martinsried, Germany; 3 Graduate School for Systemic Neuroscience, LMU Munich, 82152 Martinsried, Germany

## 

The hippocampus is a brain structure that is involved in the formation and recall of episodic memories, including space-related behavior. Place-specific hippocampal activity patterns change dramatically if the animal is exposed to the same maze in different lab environments (global remapping) [1]. Global remapping leads to decorrelated activity patterns, which is essential for many models of associative memory: it sustains flexibility and allows many patterns to be stored.

During hippocampal remapping the spatial activity pattern of grid cells undergoes locally coherent rotations and translations [2], but it is not known whether this coherence extends over different spatial modules, which are anatomically separated within the entorhinal cortex [3]. Within a computational approach, we studied the influence of the incoherent realignment of different independent modules on the formation of place cell firing patterns in the hippocampus. We find that global remapping can indeed be caused by realigning a realistic number of modules, given the experimental constraints.

**Figure 1:**
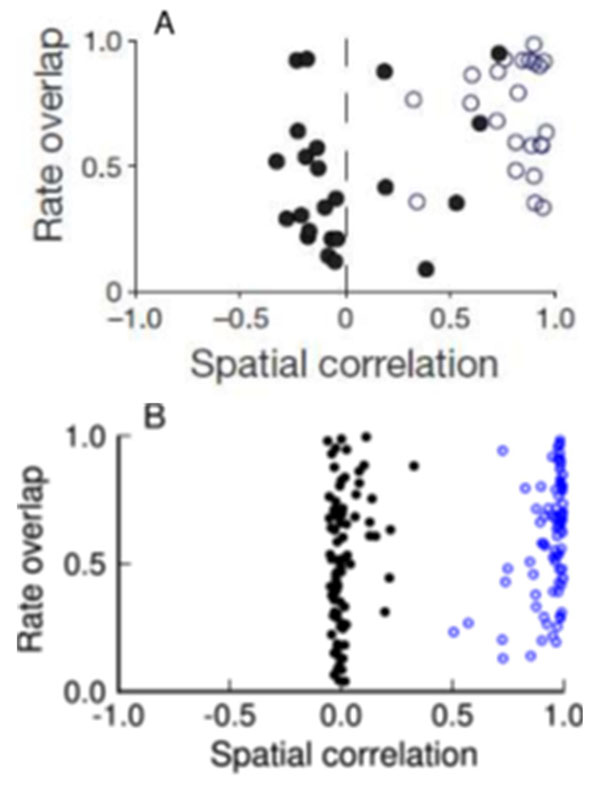
Experiments versus Theory. **A)** Figure adapted from [2]. For different environments (black dots), small spatial correlation exists between place maps, and the rates can vary drastically (as measured by the rate overlap). Returning to the initial environment (blue circles) retrieves the old map with only slight changes to the rates. **B)** In simulations, large-scale, incoherent translations and rotations (black dots) of grid modules induce global remapping. In the control condition (return to the original environment, blue dots), noise was added to the place cell firing patterns, resembling the trial-to-trial variance of experiments.

We employed a model in which a superposition of grid cell outputs give rise to place cells for a single environment [4]. We investigate the behavior of the place cell output layer in response to incoherent rotations and translations of the spatial patterns in the grid cell modules, while leaving the synaptic weights from the grid layer to place layer unaltered. Simulations of the full model qualitatively agree with the findings in experiments by Fyhn et al. 2007 (Fig. 1). For different environments, place cells are spatially uncorrelated (black dots) and rates vary strongly due to global remapping. Returning the animal to the initial environment, as a control condition, produces the old map (blue circles) with largely similar rates. In these simulations the maximal length of shifts in each module is constrained by experiments [2].

The grid layer thus provides a low dimensional parameterization of the different place maps without any synaptic plasticity in the projections, thereby indexing spatial memories. This result suggests that different independent modules are present in the entorhinal cortex and that their grid fields co-realign during global remapping.
